# Building a bright, evidence-informed future: a conversation starter from the incoming editors

**DOI:** 10.1186/s12961-017-0257-x

**Published:** 2017-10-11

**Authors:** Tari Turner, Fadi El-Jardali

**Affiliations:** 1 School of Public Health and Preventive Medicine, Monash University, Melbourne, Australia; 2 Department of Health Management and Policy, Knowledge to Policy (K2P) Center, Faculty of Health Sciences, American University of Beirut, Beirut, Lebanon

**Keywords:** Agenda setting, Capacity-building, Equity, Health research systems, Health policies, Improved health, Knowledge production and translation, Research evaluation, Research impact assessment, Sustainable Development Goals

## Abstract

Health Research and Policy Systems (*HARPS*) has gone from strength to strength since it was established in 2003. As new Editors-in-Chief, we look forward to a bright future for *HARPS*, and we would like to start a conversation with you, *HARPS* readers, authors, editors and others, about how *HARPS* can best support ongoing progress and debate on evidence-informed health research policy and systems, particularly in developing countries. As a starting point for discussion, we would like to highlight three areas that we are passionate about, namely supporting an integrated community of researchers and policy-makers; building a focus on how health research and policy systems can support achievement of the Sustainable Development Goals; and strengthening our commitment to communicating and disseminating the work published in *HARPS*. We invite you to contribute your thoughts, ideas and suggestions on the future of *HARPS*, as we work together towards an evidence-informed future.

## Editorial

*Health Research and Policy Systems* (*HARPS*) was established in 2003 and has a mandate to further “*the intellectual debate and discourse on the important role of evidence-informed health research policy and systems. It is all about sharing knowledge in order to improve health and health equity, especially in developing countries*” [1].

The progress and achievements of *HARPS* under Steve Hanney’s and Miguel Angel González Block’s leadership are impressive, with steady increases in publication numbers and impact factor, as well as strong, diverse contributors [2]. Since its inception, *HARPS* has been a vehicle and a forum for the dissemination of high quality and timely research on the role of research in strengthening health systems and improving health outcomes. It is a privilege, a pleasure and a welcome challenge to be taking on the roles of Co-Editors-in-Chief.

Nevertheless, despite the excellent work of *HARPS* and others to support the use of research in informing health practice and policy, there is a still a great deal of work to be done. In 2013, the WHO Health Report observed that: “*The long-observed gap between existing knowledge and health practice remains wide. Still greater effort is needed to translate evidence into policy and practice*” [3]. This effort needs to encompass research capacity-building, research priority-setting, knowledge translation and community building, all areas in which *HARPS* has an established track record.

Similarly, in spite of the progress that has been made in enhancing the use of research evidence in health policy decisions, a number of important methodological challenges and uncertainties remain, particularly in areas related to (1) understanding which knowledge translation strategies and tools are most effective and in what contexts and institutional supports; (2) integrating other forms of evidence into health policy-making; and (3) defining and measuring impact of knowledge translation and institutionalisation of efforts to promote evidence-informed decisions [4–7]. These too are areas that *HARPS* can build upon its existing substantial strengths.

*HARPS* aims to play a key part in the global effort to overcome the know–do gap, and we would highly appreciate your input into how to best achieve this. Our initial plans have several elements, including supporting an integrated community of researchers and policy-makers; building a focus on how health research and policy systems can support achievement of the Sustainable Development Goals (SDGs); and strengthening our commitment to communicating and disseminating the work published in *HARPS*.

## Supporting an integrated community of researchers and policy-makers working together

Health policy-making is a complex, iterative, multidimensional process to which evidence from research is one contributor [8]. In this environment, knowledge translation is most effective when all stakeholders actively contribute throughout the process, from research prioritisation to research uptake.

We believe that *HARPS* can bring all elements of the research–policy world together – such that the research which is done is useful and that it is used. To enable this, we are considering new approaches, such as bringing on board policy-makers as editors and reviewers, and reaching beyond health to support cross-sectoral and cross-disciplinary research, recognising that there is an opportunity to learn from other areas of research and policy about how to effectively bring these two worlds together. Further emphasis is also needed on approaches, frameworks and measures to strengthen health systems research and promote evidence-informed health policies, as well as innovative demonstrations of health systems impact. We welcome your suggestions on how we can support these types of undertakings.

## Building a focus on how health research and policy systems can support achievement of the SDGs

While global efforts on the Millennium Development Goals led to substantial reductions in poverty, progress was not even or necessarily equitable [9]. In the new global context, a renewed effort is needed to meet SDGs and to improve health, particularly for the poorest and most marginalised. The SDGs cover a wide range of social, environmental and economic issues. Therefore, policy decisions and action to meet them need to be informed by high quality policy-relevant evidence co-designed and co-produced with the relevant stakeholders and policy-makers, and communicated in user-friendly and timely ways, taking into consideration political context and processes. Strengthening the science–policy–society interface and ensuring the continuity of science–policy dialogue in all areas of sustainable development is imperative for all countries and at all levels [10, 11]. Progress on SDG implementation also needs to be supported by a meaningful framework and metrics for identifying, measuring and reporting on the right indicators from all sectors in reliable and valid ways. To promote multi-sectoral actions for health, the evidence should be produced in an interdisciplinary way and translated into multi-sectoral policy decisions through a process of participation and engagement by relevant stakeholders [12]. We believe this is a key opportunity for *HARPS*.

## Strengthening our commitment to communicating and disseminating the work published in *HARPS*

We know that, as a journal, *HARPS* needs to be actively disseminating research, whilst seeking dialogue with its readers, contributors, editors and others about how we can most effectively play our part in the health research and policy system.

We hope to convene conversations with researchers, health decision-makers and others. We look forward to leveraging social media platforms and launching a *HARPS* Twitter account to draw together communities of interest and to shape discussions and debate in the field of health research systems and policy. We also plan to develop, maintain and strengthen relations with a wide variety of global and regional health research and policy institutions.

We hope that these changes, and others that you propose, will have a positive impact in further developing the community of scholars, thinkers and visionaries striving to enhance the use of evidence in decision-making and closing the know–do gap. We hope you enjoy and learn from our journal, as in the past, and find it a source of up-to-date, high quality and cutting-edge information in the field of health research policy and systems.

We welcome potential contributors with original research, reviews, commentaries and opinion papers within the scope of the journal. The journal will also host special issues, as well as invited reviews and commissioned commentaries from opinion leaders. We encourage you to participate even more actively in the journal as contributors of articles, readers, reviewers and advocates for ideas in the field of health research policy and systems.

The future is bright, but there is a lot of work to be done to get us there. We look forward to working with you.

## Abbreviations

HARPS: Health Research and Policy Systems
SDGs: Sustainable Development Goals
